# Effects of butylated hydroxytoluene (BHT) metabolites on sperm quality in adult males: a cross-sectional study

**DOI:** 10.3389/frph.2026.1998792

**Published:** 2026-10-29

**Authors:** Weihong Yan, Wen Yao, Qian Xu, Yuanyi Ha, Yunfang Li, Juan Zhang

**Affiliations:** 1 Reproductive Medicine Department, General Hospital of Central Theater Command, Wuhan, China; 2 Department of Obstetrics and Gynecology, Henan Provincial People’s Hospital, Zhengzhou, China

**Keywords:** BHT metabolites, BKMR, butylated hydroxytoluene, environmental endocrine disruptors, male infertility, sperm quality

## Abstract

**Purpose:**

To investigate the associations between urinary concentrations of BHT and its metabolites and sperm quality parameters in adult males.

**Materials and Methods:**

A total of 282 adult males seeking fertility care were enrolled. Urinary concentrations of BHT metabolites (BHA, BHT-CHO, BHT-COOH, BHT-OH, BHT-Q, and BHT-quinol) were measured by HPLC MS/MS. Sperm quality parameters included sperm count, sperm concentration, progressive motility, normal morphology rate, and DNA fragmentation index (DFI). Multiple linear regression and Bayesian kernel machine regression (BKMR) were used to evaluate individual and joint associations, adjusting for age, BMI, household income, and smoking status.

**Results:**

In multiple linear regression, higher tertiles of urinary BHA were significantly positively associated with higher sperm concentration (*P* for trend < 0.05). Similarly, higher tertiles of BHT-COOH were significantly positively correlated with more total sperm count (*P* for trend < 0.05). Higher tertiles of BHT-OH, BHT-Q, and BHT- quinol were significantly positively associated with normal sperm morphology rate (all *P* for trend < 0.05). BKMR model did not reveal a monotonic overall joint effect of the metabolite mixture on total sperm count, sperm concentration, or normal morphology rate, but individual metabolite trends were consistent with the linear regression results.

**Conclusions:**

The overall elevated concentrations of BHT and its metabolites showed no significant correlation with sperm quality, while increased levels of certain metabolites were positively associated with improved sperm quality. Further in-depth investigations are still required to systematically clarify the effects of BHT on sperm quality, so as to provide solid scientific evidence for the comprehensive improvement of male reproductive health.

## 1 Introduction

Globally, the prevalence of infertility is reported to be 15%, with male factors accounting for 40%–50% of cases (1). In China, an epidemiological survey based on 18,571 couples across eight provinces indicated that the prevalence of infertility among couples of reproductive ages is 25 percent (2). Sperm quality is a critical determinant of infertility. From 1973 to 2018, the global mean sperm concentration declined by 51.6%, and the rate of decline accelerated further after 2,000, reaching 2.64% annually (3). This trend is not localized but represents a widespread issue across multiple regions, suggesting that the male reproductive system may be increasingly affected by persistent and extensive external factors. Despite existing intervention programs targeting male reproductive health, the problem of male infertility remains unresolved, largely due to the continued presence of adverse factors (4). These factors may impair spermatogenesis and sperm function through endocrine disruption and oxidative stress, with the demand of improving male reproductive health becoming increasingly urgent. Therefore, elucidating the mechanisms by which lifestyle and environmental factors affect sperm quality and identifying effective interventions are of critical importance (5, 6).

Environmental pollutants, as exogenous risk factors that cannot be overlooked, are posing an increasingly significant potential threat to the male reproductive system as the pace of industrialization and urbanization accelerates. The environment is now replete with various pollutants that can impair sperm quality. The U.S. Environmental Protection Agency (EPA) defines endocrine disrupting compounds as “substances that interfere with the synthesis, secretion, transport, binding, or elimination of natural hormones that regulate homeostasis, reproduction, development and behavior.” These include heavy metals, pesticide residues, and plastic additives, amongst others (7). For instance, lead can cross the blood–testis barrier to enter testicular tissue, affecting the activity of testosterone synthase, reducing testosterone levels, which also damage to sperm DNA and increase the rate of chromosomal aberrations in sperm (8). These substances represent a persistent threat to male reproductive health via air, water, food, soil, etc (9, 10).

In recent years, synthetic phenolic antioxidants (SPAs) have attracted widespread attention. SPAs are widely used in industrial production and food processing to inhibit oxidative deterioration, which are commonly applied to edible oils, fried foods, instant noodles, and other food products to extend their shelf life, also prevent material ageing in products such as soap and plastic packaging (11).

Butylated hydroxytoluene (BHT), a typical SPAs, is thought to impair sperm quality mainly through three pathways: disruption of endocrine balance, triggering oxidative stress, and damaging to cellular genetic material, which effects are cumulative and insidious (12). Animal studies have shown that long term exposure to BHT significantly reduces male reproductive capacity, manifested by decreasing sperm concentration and motility (13). Whereas, direct epidemiological data in humans are still lacking. To investigate the effects of BHT and its metabolites on sperm quality, this study, enrolling 282 adult males, analyzed the association between urinary metabolites and sperm quality with multiple linear regression and Bayesian Kernel Machine Regression (BKMR) model.

## 2 Patients and methods

### 2.1 Study subjects

A total of 321 adult males seeking fertility care at a reproductive medicine center were initially screened. Eligible participants were those who voluntarily agreed to participate and were able to provide complete questionnaires, serum samples, and semen samples.

*Inclusion criteria:*

1) Adult males age 20–45 years.
2) Individuals plan to conceive with their spouses and receive fertility consultation.
3) Individuals can comply with sample collection and questionnaire completion.

*Exclusion criteria:*

1) Individuals with congenital reproductive system malformations, trauma to the reproductive tract, or severe systemic diseases, such as severe liver or kidney dysfunction, endocrine disorders, etc.
2) Individuals with a history of chemotherapy or radiotherapy, or long-term use of medications affecting reproductive function.
3) Individuals who have undergone major surgery or suffered from severe infectious disease within the previous 3 months.
4) Individuals with improper semen sample collection or missing test data

### 2.2 Sample size estimation

In this study, indicators of male fertility, such as sex hormone levels and sperm motility, were all continuous variables, necessitating the use of generalized linear models. Therefore, the *F* test was employed for sample size calculation. Assuming that environmental endocrine disruptors caused 10% difference, with a statistical power of 80% and a significance level of 0.05, consequently the required minimum sample size was 143.The study was adequately powered.

### 2.3 Urine sample collection and detection of environmental endocrine disruptors

Urine sample collection: On the same day as semen collection, a random urine sample (10–15 mL) was obtained from each participant in a sterile cup. The samples were promptly transferred to a centrifuge tube and centrifuged at 3,000 r/min for 10 min, and the supernatant stored in a freezer at −80 °C for subsequent analysis.

Detection of environmental endocrine disruptors: Urinary concentrations of BHT metabolites (BHA, BHT-CHO, BHT-COOH, BHT-OH, BHT-Q, and BHT-quinol) were detected by High-Performance Liquid Chromatography-Tandem Mass Spectrometry (HPLC MS/MS).

### 2.4 Semen sample collection and sperm quality analysis

After a recommended abstinence period of 2–7 days, the subjects collected semen samples by masturbation into sterile, clean collection cups. The semen samples were liquefied for 30 min in an incubator maintained at 37 °C. Sperm quality parameters, including sperm count, sperm concentration, and progressive motility, were assessed using a computer assisted semen analysis system (Olympus). Each semen smear was analyzed by examining at least 200 spermatozoa, followed by calculation of the proportion of morphologically normal spermatozoa.

The DNA Fragmentation Index (DFI) was measured with a sperm DNA fragmentation kit (Anke Biotechnology) according to the manufacturer's instructions, and calculated by the following formula: (fragmented sperm count+degraded sperm count)/total counted sperm   ×   100%.

### 2.5 Questionnaire survey

A self-designed questionnaire was administered through face-to-face interviews by trained research staff. The questionnaire covered:

1) Basic demographic characteristics: age, ethnicity, height, weight, Body mass index (BMI), educational level, and household income and so on.
2) Lifestyle habits: smoking status and alcohol consumption status, for example, never, currently, or abstained for over one year.

### 2.6 Statistical analysis

#### 2.6.1 Descriptive statistics analysis

For continuous variables, normally distributed data were expressed as mean ± standard deviation (x¯ ± s), and non-normally distributed data as median (interquartile range) [M (P_25_, P_75_)]. And the categorical variables were presented as counts (percentages) [n (%)]. Besides, Missing baseline information was imputed using the mean value.

#### 2.6.2 Multiple linear regression analysis

After logarithmic transformation of the urinary concentrations of BHT and its metabolites, they were divided into three tertiles (Q1-Q3) to reduce bias. Multiple linear regression models were applied to analyze the associations between sperm quality parameters and BHT and its metabolites. The trend tests were performed by entering tertiles as ordinal categorical variables (1–3) into the models. Adjusted covariates included age, BMI, household income, smoking status, and alcohol consumption status, etc.

#### 2.6.3 BKMR analysis

The BKMR model was employed to evaluate the combined effects of multiple environmental endocrine disruptors on sperm quality. Posterior inclusion probabilities (PIPs) were calculated to reflect the importance of individual metabolites. Metabolite levels and continuous covariates (age, BMI) were standardized. Sperm quality parameters were assumed to follow a Gaussian distribution. The model was run for 50,000 iterations, with the first 25,000 iterations used as the burn-in phase.

### 2.7 Ethical statement

The study protocol was approved by the institutional review board of the General Hospital of Central Theater Command, Wuhan, China [approval No. (2023)087-01]. Informed consent was confirmed by the IRB.

## 3 Result

### 3.1 Baseline characteristics

This study ultimately included 282 adult males, recruiting from individuals seeking fertility treatment at reproductive medicine department of General Hospital of Central Theater Command between December 2023 and June 2024. Baseline characteristics are summarized in Table 1.

1) Demographic characteristics: The average age of the study participants was 30.7 years, with an average height of 173.5 cm, an average weight of 72.3 kg and an average BMI of 24.0 kg/m^2^. The majority of participants were of Han ethnicity, accounting for approximately 94.0%, whilst a small proportion belong to other ethnicity, accounting for 6.0%. Regarding educational level, 232 subjects were university graduate or higher, accounting for 82.3%, others were the high school graduate or below about 17.7%. Besides, 42.9% of participants had a monthly household income of ≤10,000 yuan per month, and 57.1% had an income of >10,000 yuan per month. With respect to smoking status, 36.9% of participants were smokers and the others were non-smokers. Regarding alcohol consumption, merely 15 participants were drinkers accounting for about 5.3% and most of them were non-drinkers. There was a missing measurement for the height of one male, and we used the average value (173.5 cm) to replace it.
2) Reproductive parameters: The mean total sperm count in the participants’ semen was 685.3 ± 516.2 million, and the mean sperm concentration was 162.7 ± 113.6 million/mL. Then the mean progressive motility was 37.7 ± 15.5%, and the mean normal morphology rate was 4.4 ± 1.6%, simultaneous the mean DFI was 15.3 ± 5.5%. Nevertheless, there are 16 male subjects have missing morphological data and 30 male subjects have missing DFI data. Such records were omitted from the analytical dataset since the outcome variables could not be imputed.

**Table 1:** Characteristics of the 282 men included in this study^a^.

Parameter	Mean ± SD or N (%)
Basic information	
Age (years)	30.7 ± 3.7
Height (cm)	173.5 ± 5.5
Weight (kg)	72.3 ± 9.6
BMI (kg/m^2^)	24.0 ± 2.7
Abstinence times (days)	5.6 ± 4.2
Ethnicity	
Han	265 (94.0%)
Other ethnic minorities ^b^	17 (6.0%)
Educational level	
High school and below	50 (17.7%)
University and above	232 (82.3%)
Monthly household income (yuan/month)	
≤10000 (reference)	121 (42.9%)
>10000	161 (57.1%)
Smoking status	
No(reference)	178 (63.1%)
Yes	104 (36.9%)
Alcohol consumption status	
No(reference)	267 (94.7%)
Yes	15 (5.3%)
Clinical characteristics	
Sperm count (million)	685.3 ± 516.2
Sperm concentration (million/mL)	162.7 ± 113.6
Progressive motility (%)	37.7 ± 15.5
Normal sperm morphology rate (%)	4.4 ± 1.6
DFI (%)	15.3 ± 5.5

a Missing data: height for 1 male; normal sperm morphology rate for 16 males; DFI for 30 males.

b Other ethnic minorities include: the Dong, Hui, Gelao, Lisu, Mongol, Tujia and Zhuang.

BMI, body mass index; SD, standard deviation; DFI, DNA Fragmentation Index.

### 3.2 Urinary concentrations of BHT and its metabolites

The distribution of the nine measured environmental endocrine disruptor metabolites in urine was skewed; therefore, geometric means were used as measures of central tendency. Among the BHT metabolites, BHT-Q showed the highest concentration, reaching 2,406.6 ng/mL, whereas BHT-quinol revealed the lowest concentration, at 24.1 ng/mL (Table 2).

**Table 2:** Distribution of metabolite concentrations in urine.

Metabolite (ng/mL)	AM	GM	Percentile		
			25%	50%	75%
BHA	82.2	24.7	8.3	22.8	59.4
BHT-CHO	266.8	69.5	31.5	84.2	168.8
BHT-COOH	128.8	49.1	23.1	54.4	131.1
BHT-OH	121.0	29.4	8.4	21.3	86.3
BHT-Q	24473.0	2406.6	1143.0	2831.0	8470.0
BHT-quinol	112.6	24.1	7.0	16.3	84.2

AM, Arithmetic Mean; GM, Geometric mean.

### 3.3 Association of individual BHT and metabolites concentrations with sperm quality

Multiple linear regression models were used to analyzed the associations between individual urinary metabolites concentrations and sperm quality parameters (total sperm count, sperm concentration, progressive motility, normal sperm morphology rate, and DFI). The models were adjusted for covariates covering age, BMI, household income, and smoking status etc. The results are presented in Table 3. Higher tertiles of urinary BHA were significantly positively associated with higher sperm concentration (*P* for trend < 0.05). Similarly, higher tertiles of BHT-COOH were significantly positively correlated with more total sperm count (*P* for trend < 0.05).   . Higher tertiles of BHT-OH, BHT-Q, and BHT- quinol were significantly positively associated with normal sperm morphology rate (all *P* for trend < 0.05).

**Table 3:** Association of individual BHT and metabolites concentrations with sperm quality.

BHT Metabolites	Sperm count	Sperm concentration	Progressive motility	Normal sperm morphology rate	DFI
	Change% (95% CI)	Change% (95% CI)	Change% (95% CI)	Change% (95% CI)	Change%(95% CI)
BHA					
Q1(<11.1)	ref	ref	ref	ref	ref
Q2 (11.1–40.9)	**188.9 (42.6, 335.3)**	30.6 (–1.9, 63.0)	0.2 (–4.2, 4.6)	–0.2 (–0.7, 0.2)	1.2 (–0.5, 2.9)
Q3(>40.9)	128.2 (–18.8, 275.1)	**34.2 (1.7, 66.8)**	3.8 (–0.6, 8.2)	0.1 (–0.4, 0.5)	–0.5 (–2.2, 1.2)
*P* for trend	**0.09**	**0.04**	**0.09**	0.84	0.54
BHT-CHO					
Q1(<52.6)	ref	ref	ref	ref	ref
Q2 (52.6–132.2)	75.2 (–72.6, 223.0)	20.5 (–12.1, 53.1)	1.8 (–2.7, 6.2)	–0.1 (–0.6, 0.4)	–0.8 (–2.5, 0.9)
Q3(>132.2)	–3.8 (–151.7, 144.0)	–4.6 (–37.2, 28.0)	2.2 (–2.2, 6.7)	0.2 (–0.3, 0.7)	–0.7 (–2.4, 1.0)
*P* for trend	**0.09**	0.78	0.32	0.44	0.39
BHT-COOH					
Q1(<33.0)	ref	ref	ref	ref	ref
Q2 (33.0–98.3)	65.0 (–82.3, 212.3)	13.3 (–19.3, 46.0)	0.2 (–4.2, 4.6)	–0.1 (–0.6, 0.4)	0.9 (–0.8, 2.6)
Q3(>98.3)	**154.3 (5.1, 303.5)**	28.8 (–4.3, 61.8)	–0.7 (–5.2, 3.8)	–0.3 (–0.8, 0.2)	1.6 (–0.1, 3.3)
*P* for trend	**0.04**	**0.09**	0.76	0.26	**0.07**
BHT-OH					
Q1(<10.1)	ref	ref	ref	ref	ref
Q2 (10.1–59.9)	–62.5 (–210.8, 85.8)	–8.7 (–41.5, 24.1)	3.23 (–1.2, 7.7)	**0.8 (0.3, 1.3)**	–0.1 (–1.8, 1.6)
Q3(>59.9)	29.7 (–117.6, 177.0)	5.9 (–26.7, 38.5)	–0.2 (–4.6, 4.2)	**0.7 (0.3, 1.2)**	0.1 (–1.6, 1.8)
*P* for trend	0.69	0.72	0.93	**<0.01**	0.89
BHT-Q					
Q1(<1537.0)	ref	ref	ref	ref	ref
Q2 (1537.0–5435.0)	6.2 (–143.0, 155.43)	10.7 (–22.2, 43.6)	–1.8 (–6.2, 2.7)	0.4 (–0.1, 0.8)	–0.6 (–2.3, 1.1)
Q3(>5435.0)	–7.2 (–155.0, 140.7)	–7.3 (–39.9, 25.3)	1.2 (–3.2, 5.6)	**0.5 (0.1, 1.0)**	0.4 (–1.3, 2.1)
*P* for trend	0.92	0.66	0.59	**0.03**	0.66
BHT-quinol					
Q1(<8.7)	ref	ref	ref	ref	ref
Q2 (8.7–35.6)	95.3 (–52.5, 243.1)	11.5 (–21.2, 44.3)	–1.1 (–5.5, 3.4)	**0.5 (0.0, 0.9)**	–0.2 (–1.9, 1.5)
Q3(>35.6)	33.3 (–115.3, 181.9)	3.4 (–29.5, 36.3)	0.3 (–4.2, 4.7)	**0.5 (0.1, 1.0)**	0.5 (–1.2, 2.2)
*P* for trend	0.65	0.84	0.91	**0.03**	0.58

The linear regression model has been adjusted for age, BMI, household income and smoking status. The *P*-value for trend was obtained by entering the tertiles of the exposure factors as an ordinal categorical variable (1–3) in the analysis. Q1, Q2, and Q3 respectively represent the first, second, and third tertiles of the urinary concentrations of BHT and its metabolites. BMI, body mass index; SD, standard deviation; DFI, DNA Fragmentation Index.

### 3.4 Associations of multiple BHT metabolites with sperm quality

The joint effects of BHT related metabolites (BHA, BHT-CHO, BHT-COOH, BHT-OH, BHT-Q, and BHT- quinol) on sperm quality parameters were evaluated with BKMR model.

1) Overall joint effect: The BKMR model indicated a monotonic increase in total sperm count, sperm concentration, or normal morphology rate with rising concentrations of the BHT-related metabolites (Figure 1).
2) Group-specific contribution differences: BHA and BHT-Q exhibited higher group PIPs, suggesting a potentially greater contribution to the association between BHT metabolites and sperm quality (Table 4).
3) Trend Analysis of individual metabolites: In the BKMR model, trends consistent with the multiple linear regression results were observed. That is to say, increasing BHA concentration was relevant to a steady increase in total sperm count, sperm concentration and progressive motility. Likewise, as BHT-OH concentration increases, the normal morphology rate shows an upward trend (Figure 2).

**Figure 1:**
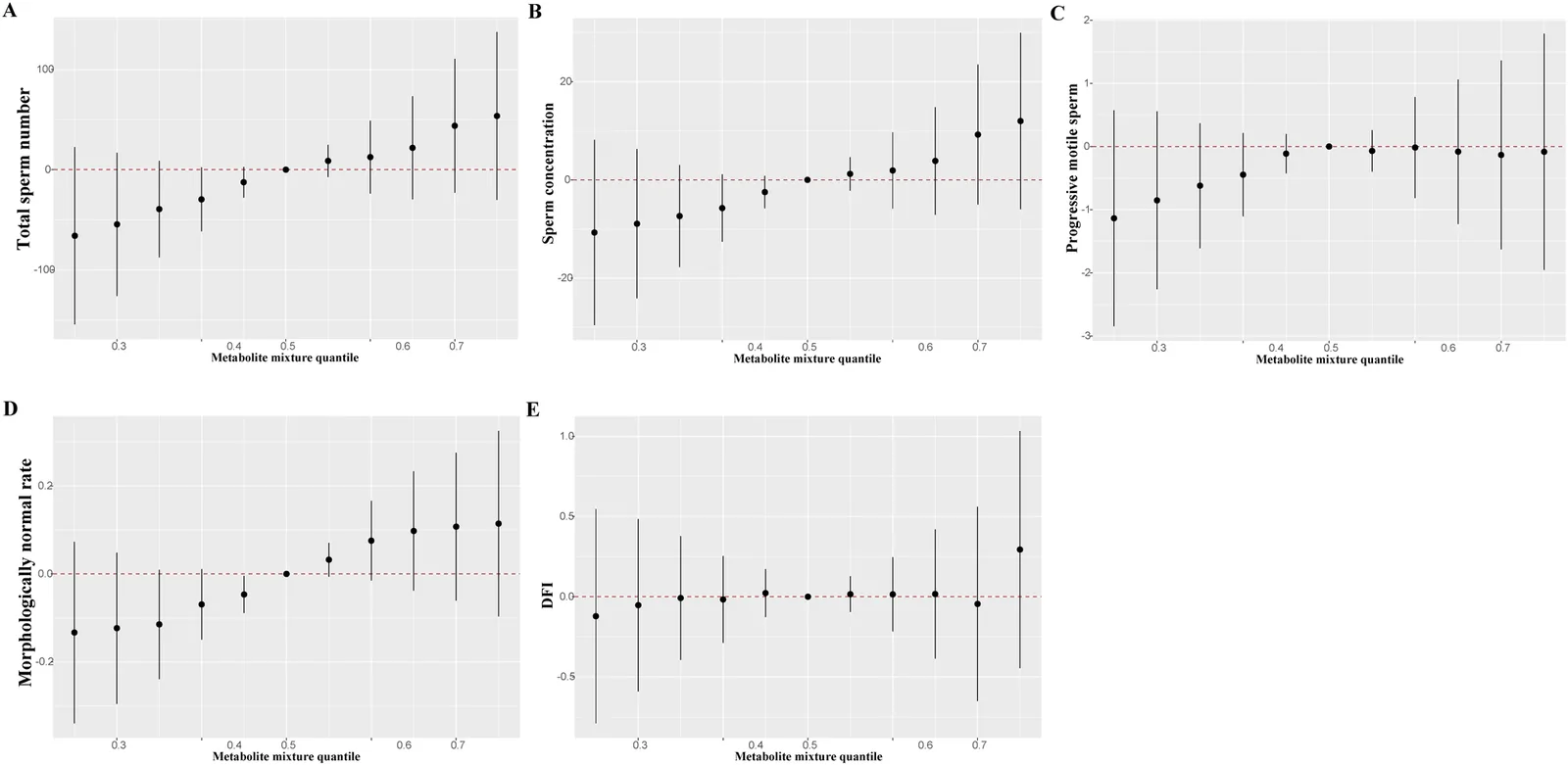
Effect size of BHT metabolites mixture on sperm quality parameters analyzed by the BKMR model (95% confidence intervals). Sperm quality parameters:**(A)** Sperm count; **(B)** Sperm concentration; **(C)** Progressive motility; **(D)** Normal sperm morphology rate; **(E)** DFI. Compare the concentration levels of all metabolites at a specific percentile with those at the 50th percentile. BKMR model has been adjusted for age, body mass index (BMI), household income and smoking status.

**Table 4:** PIPs for sperm quality parameters.

BHT Metabolites	Sperm count	Sperm concentration	Progressive motility	Normal sperm morphology rate	DFI
BHA	0.47	0.46	0.17	0.06	0.10
BHT-CHO	0.26	0.33	0.04	0.03	0.06
BHT-COOH	0.26	0.32	0.04	0.09	0.10
BHT-OH	0.28	0.29	0.09	0.60	0.11
BHT-Q	0.44	0.42	0.14	0.03	0.05
BHT-quinol	0.32	0.34	0.06	0.18	0.12

BKMR model has been adjusted for age, BMI, household income, and smoking status. PIPs, posterior inclusion probabilities; BMI, body mass index; BKMR, Bayesian kernel machine regression; DFI, DNA Fragmentation Index.

**Figure 2:**
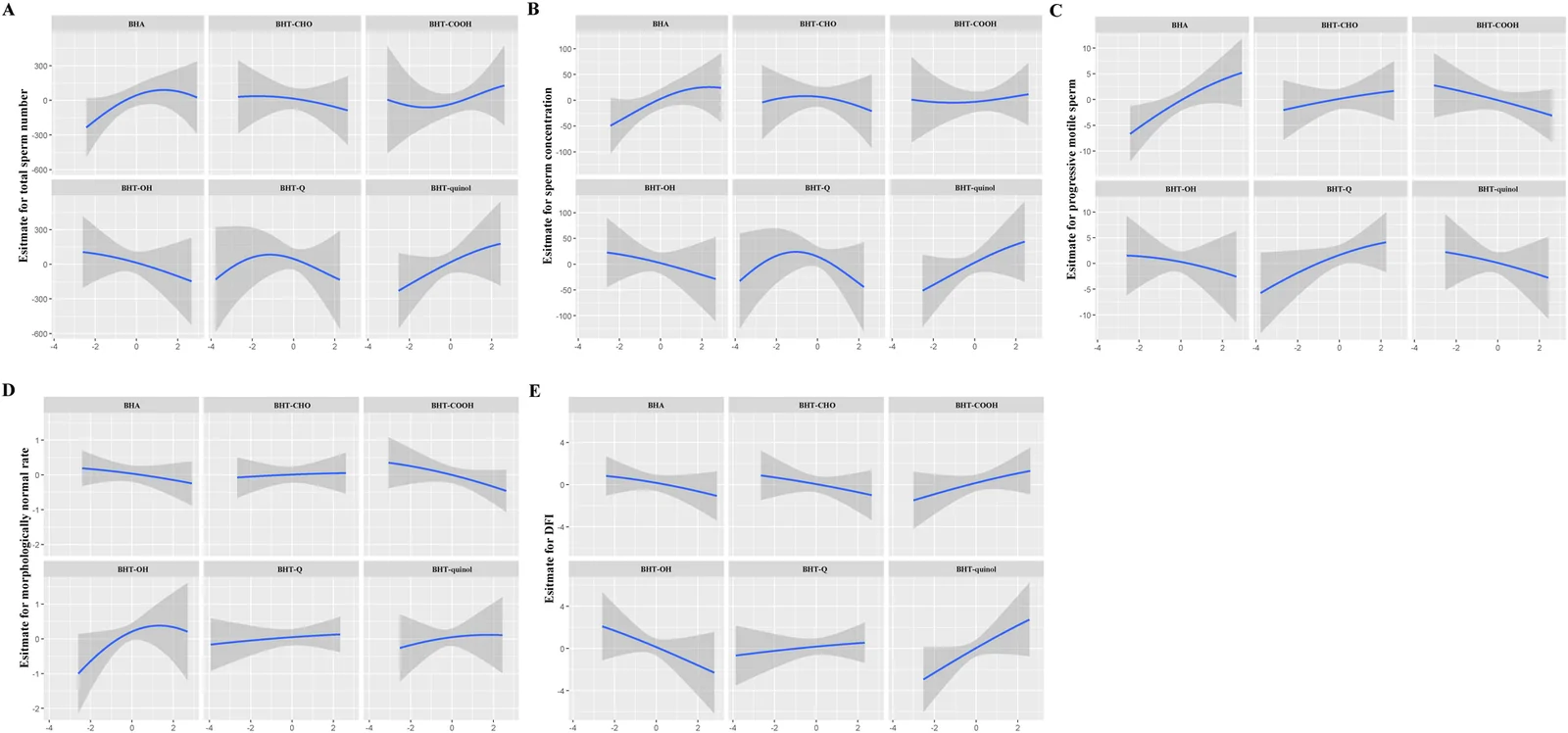
Univariate exposure-response relationships between the concentrations of various metabolites and sperm quality parameters, with 95% confidence intervals, when the concentration of other BHT metabolites is fixed at the median value. Sperm quality parameters:**(A)** Sperm count; **(B)** Sperm concentration; **(C)** Progressive motility; **(D)** Normal sperm morphology rate; **(E)** DFI. BKMR model has been adjusted for age, body mass index (BMI), household income and smoking status.

## 4 Discussion

### 4.1 Exposure characteristics of BHT metabolites

BHT and its metabolites are widely used as synthetic antioxidants in food packaging, cosmetics, and other products, primarily to inhibit lipid oxidation, delay food spoilage, and extend shelf life. Exposure to these substances is widespread; they can be absorbed by the human body via the digestive tract and the skin, amongst other routes. Following metabolism in the liver and kidneys, they are primarily excreted in urine as characteristic metabolites including BHT-CHO, BHT-COOH, BHT-OH, BHT-Q, and BHT-quinol (11). Although Chinese national standards stipulate that the total content of BHT and BHA, when used in combination, should not exceed 100 mg/kg. Beyond that the total amount of BHT, whether used alone or in combination, must comply with the overall SPAs limit of 200 mg/kg, however, some studies have reported excessive levels of BHT in food products. For example, Yang et al. detected 17 edible oil samples and discovered that in four of them, the total concentrations of BHT and other SPAs ranging from 235.1 to 435.4 mg/kg, far exceeding the regulatory limits, which was attributed to overuse by manufacturers to prolong shelf life (14). Similarly, high urinary concentrations of BHT have been detected in the samples from Japan, India, the United States, and other countries (15).

### 4.2 Comparison with previous studies

To our knowledge, no epidemiological study has directly examined the association between urinary BHT exposure levels and human sperm quality. However, some experimental studies have shown that the addition of BHT as a supplement to frozen thawed semen can preserve sperm DNA integrity and reduce reactive oxygen species (ROS) production (16, 17). These results are consistent with the positive associations that observed between BHA, BHT-COOH, BHT-OH, BHT-Q, and BHT-quinol and several sperm quality parameters. In contrast, a case–control study conducted in China manifested that BHT and its metabolites could cross the blood–follicle barrier into follicular fluid, which high concentrations of BHT-CHO, BHT-COOH, and BHT-Q in follicular fluid were significantly associated with an increased risk of diminished ovarian reserve (18). This discrepancy may be related to sex specific differences in the distribution and metabolism of BHT related compounds between follicular fluid and urine. In animal experimental studies, feeding mice with 0%-0.405% BHT over three generations had little adverse effect on reproductive and neurobehavioral parameters (19). Nevertheless, an *in vitro* study demonstrated that exposure of mouse testicular Leydig cells (TM3) and Sertoli cells (TM4) to 0–100*μ*M BHT for 24 h induced calcium homeostasis disruption, endoplasmic reticulum mitochondrial dysfunction, and suppressed steroidogenesis related gene expression, ultimately resulting in testicular dysfunction (20). Given the relatively limited evidence on the reproductive effects of BHT, further clinical and basic research is needed to elucidate its impact on reproductive function and the underlying mechanisms.

### 4.3 Potential mechanisms

BHT is widely used as an antioxidant in the food and industrial sectors, one of which core mechanisms of action is likely related to the regulation of oxidative stress by scavenging free radicals, inhibiting lipid peroxidation, and reducing excessive ROS accumulation, thereby maintaining redox balance (21). Whereas exposure of zebrafish embryos to BHT resulted in increased ROS generation and elevated 8-hydroxy-2′-deoxyguanosine (8-OHdG), a marker of DNA oxidative damage, leading to embryotoxicity (22). In male clams exposed to BHT, meiosis and spermatogenesis were inhibited, which generated ROS levels increasing in the testes, reducing mature sperm counts, and a lower gonadosomatic index (23). It is speculated that this discrepancy may be related to the dose used. The environmentally relevant BHT concentrations may be low, whereas the experimental interventions often employ high doses that disrupt redox homeostasis and promote ROS production.

Likewise，discrepancies between animal toxicology data and our human cross-sectional results may be partially explained by non-monotonic dose–response relationships (24). In experimental animal studies, BHT is typically administered at relatively high doses that elicit adverse reproductive outcomes, whereas human exposure in the general population occurs at far lower environmental levels. Non-monotonic effects are widely reported for endocrine-disrupting chemicals: low concentrations may trigger adaptive or stimulatory biological responses, while high doses produce toxicity (25, 26).

The precise mechanisms by which BHT and related endocrine disruptors induce reproductive toxicity remain largely unclear, and further studies are warranted in future.

### 4.4 Strengths and limitations

This study utilized the BKMR model to analyze the joint effects and quantified the contribution weights of different exposure categories with PIPs, thereby overcoming the limitations of traditional regression models in handling multicollinearity and providing visual evidence for dose–response relationships.

Several limitations should be acknowledged. First of all, the cross-sectional study design is unable to determine the causal relationship, then, the relatively small sample size and single center recruitment reduce the persuasiveness of the study results. And further prospective studies should to continue with larger-scale and multiple tests. Secondly, the study did not incorporate other relevant variables that might affect sperm quality, such as genetic testing, namely chromosomal karyotyping and Y-chromosome microdeletion testing, all of which undermine the reliability of the conclusions. Thirdly, exposure assessment is subject to temporal bias, as urinary metabolites only reflect recent exposure over approximately the preceding 24 h and fail to capture the dynamic relationship between long-term exposure and reproductive function.

## 5 Conclusions

On the whole, overall higher concentrations of BHT and its metabolites were not associated with impaired sperm quality but certain individual metabolites showed positive associations. Future studies are expected to utilize multi-omics technologies and longitudinal cohort study designs to establish a comprehensive research framework encompassing risk identification, mechanism elucidation, intervention implementation and outcome evaluation. Such efforts will provide more robust scientific support for comprehensively improving male reproductive health, reducing the risk of infertility and strengthening the foundations of population health, sequentially contributing to sustainable development in the field of reproductive health.

## Data Availability

The original contributions presented in the study are included in the article/Supplementary Material, further inquiries can be directed to the corresponding author.

## References

[1] Martínez MÁ Marquès M Salas-Huetos A Babio N Domingo JL Salas-Salvadó J. Lack of association between endocrine disrupting chemicals and male fertility: a systematic review and meta-analysis. Environ Res. (2023) 217:114942. 10.1016/j.envres.2022.114942 36436552

[2] Zhou Z Zheng D Wu H Li R Xu S Kang Y. Epidemiology of infertility in China: a population-based study. BJOG. (2018) 125:432–41. 10.1111/1471-0528.14966 29030908

[3] Levine H Jørgensen N Martino-Andrade A Mendiola J Weksler-Derri D Jolles M. Temporal trends in sperm count: a systematic review and meta-regression analysis of samples collected globally in the 20th and 21st centuries. Hum Reprod Update. (2023) 29:157–76. 10.1093/humupd/dmac035 36377604

[4] De Jonge CJ Barratt CLR Aitken RJ Anderson RA Baker P Chan DYL. Current global status of male reproductive health. Hum Reprod Open. (2024) 2024:hoae017. 10.1093/hropen/hoae017 38699533 PMC11065475

[5] Nordkap L Joensen UN Blomberg Jensen M Jørgensen N. Regional differences and temporal trends in male reproductive health disorders: semen quality may be a sensitive marker of environmental exposures. Mol Cell Endocrinol. (2012) 355:221–30. 10.1016/j.mce.2011.05.048 22138051

[6] Di Nisio A Foresta C. Water and soil pollution as determinant of water and food quality/contamination and its impact on male fertility. Reprod Biol Endocrinol. (2019) 17:4. 10.1186/s12958-018-0449-4 30611299 PMC6321708

[7] Kavlock RJ Daston GP DeRosa C Fenner-Crisp P Gray LE Kaattari S. Research needs for the risk assessment of health and environmental effects of endocrine disruptors: a report of the U.S. EPA-sponsored workshop. Environ Health Perspect. (1996) 104:715–40. 10.1289/ehp.96104s4715 8880000 PMC1469675

[8] Li C Zhao K Zhang H Liu L Xiong F Wang K. Lead exposure reduces sperm quality and DNA integrity in mice. Environ Toxicol. (2018) 33:594–602. 10.1002/tox.22545 29446210

[9] Zheng S Zhao N Lin X Jiang L Qiu C Jiang J. Fine particulate matter (PM2.5) and the blood-testis barrier: an *in vivo* and *in vitro* mechanistic study. Environ Health Perspect. (2025) 133:47006. 10.1289/EHP14447 40043229 PMC12010934

[10] Wang C Yang L Wang S Zhang Z Yu Y Wang M. The classic EDCs, phthalate esters and organochlorines, in relation to abnormal sperm quality: a systematic review with meta-analysis. Sci Rep. (2016) 6:19982. 10.1038/srep19982 26804707 PMC4726156

[11] Liu R Mabury SA. Synthetic phenolic antioxidants: a review of environmental occurrence, fate, human exposure, and toxicity. Environ Sci Technol. (2020) 54:11706–19. 10.1021/acs.est.0c05077 32915564

[12] Yehye WA Rahman NA Ariffin A Abd Hamid SB Alhadi AA Kadir FA. Understanding the chemistry behind the antioxidant activities of butylated hydroxytoluene (BHT): a review. Eur J Med Chem. (2015) 101:295–312. 10.1016/j.ejmech.2015.06.026 26150290

[13] Takahashi O Oishi S. Male reproductive toxicity of four bisphenol antioxidants in mice and rats and their estrogenic effect. Arch Toxicol. (2006) 80:225–41. 10.1007/s00204-005-0033-5 16231125

[14] Yang MH Lin HJ Choong YM. A rapid gas chromatographic method for direct determination of BHA, BHT and TBHQ in edible oils and fats. Food Res Int. (2002) 35:627–33. 10.1016/s0963-9969(01)00164-8

[15] Wang W Kannan K. Quantitative identification of and exposure to synthetic phenolic antioxidants, including butylated hydroxytoluene, in urine. Environ Int. (2019) 128:24–9. 10.1016/j.envint.2019.04.028 31029976 PMC6526070

[16] Ghorbani M Amiri I Khodadadi I Fattahi A Atabakhsh M Tavilani H. Influence of BHT inclusion on post-thaw attributes of human semen. Syst Biol Reprod Med. (2015) 61:57–61. 10.3109/19396368.2014.968267 25285571

[17] Merino O Aguagüiña WE Esponda P Risopatrón J Isachenko E Isachenko V. Protective effect of butylated hydroxytoluene on sperm function in human spermatozoa cryopreserved by vitrification technique. Andrologia. (2015) 47:186–93. 10.1111/and.12246 24612426

[18] Hao Y Wang Y Yan L Xu X Chen D Zhao Y. Synthetic phenolic antioxidants and their metabolites in follicular fluid and association with diminished ovarian reserve: a case-control study. Environ Health Perspect. (2023) 131:67005. 10.1289/EHP11309 37267061 PMC10237312

[19] Tanaka T Oishi S Takahashi O. Three generation toxicity study of butylated hydroxytoluene administered to mice. Toxicol Lett. (1993) 66:295–304. 10.1093/mutage/gex011 8475510

[20] Ham J Lim W Whang KY Song G. Butylated hydroxytoluene induces dysregulation of calcium homeostasis and endoplasmic reticulum stress resulting in mouse leydig cell death. Environ Pollut. (2020) 256:113421. 10.1016/j.envpol.2019.113421 31677866

[21] Xu X Liu A Hu S Ares I Martínez-Larrañaga M-R Wang X. Synthetic phenolic antioxidants: metabolism, hazards and mechanism of action. Food Chem. (2021) 353:129488. 10.1016/j.foodchem.2021.129488 33714793

[22] Baran A Yildirim S Ghosigharehaghaji A Bolat İ Sulukan E Ceyhun SB. An approach to evaluating the potential teratogenic and neurotoxic mechanism of BHA based on apoptosis induced by oxidative stress in zebrafish embryo (danio rerio). Hum Exp Toxicol. (2021) 40:425–38. 10.1177/0960327120952140 32909836

[23] Zheng X Li X Qi R Li Z Liao Q Pan L. Mechanism of reproductive toxicity of BHT on male ruditapes philippinarum: epigenetic - testis development and oxidative damage - cell death. Aquat Toxicol. (2025) 289:107590. 10.1016/j.aquatox.2025.107590 41046803

[24] Vandenberg LN Colborn T Hayes TB Heindel JJ Jacobs DR Lee D-H. Hormones and endocrine-disrupting chemicals: low-dose effects and nonmonotonic dose responses. Endocr Rev. (2012) 33:378–455. 10.1210/er.2011-1050 22419778 PMC3365860

[25] Vandenberg LN. Non-monotonic dose responses in studies of endocrine disrupting chemicals: bisphenol a as a case study. Dose Response. (2014) 12:259–76. 10.2203/dose-response.13-020.Vandenberg 24910584 PMC4036398

[26] Duh-Leong C Duh-Leong C Maffini MV Maffini MV Kassotis CD Kassotis CD. The regulation of endocrine-disrupting chemicals to minimize their impact on health. Nat Rev Endocrinol. (2023) 19:600–14. 10.1038/s41574-023-00872-x 37553404

